# Toward the use of precision medicine for the treatment of head and neck squamous cell carcinoma

**DOI:** 10.18632/oncotarget.13798

**Published:** 2016-12-04

**Authors:** Wang Gong, Yandi Xiao, Zihao Wei, Yao Yuan, Min Qiu, Chongkui Sun, Xin Zeng, Xinhua Liang, Mingye Feng, Qianming Chen

**Affiliations:** ^1^ State Key Laboratory of Oral Diseases, West China Hospital of Stomatology, Sichuan University, Chengdu, Sichuan, China

**Keywords:** big data, precision medicine, targeted therapy, gene therapy, HNSCC

## Abstract

Precision medicine is a new strategy that aims at preventing and treating human diseases by focusing on individual variations in people's genes, environment and lifestyle. Precision medicine has been used for cancer diagnosis and treatment and shows evident clinical efficacy. Rapid developments in molecular biology, genetics and sequencing technologies, as well as computational technology, has enabled the establishment of “big data”, such as the Human Genome Project, which provides a basis for precision medicine. Head and neck squamous cell carcinoma (HNSCC) is an aggressive cancer with a high incidence rate and low survival rate. Current therapies are often aggressive and carry considerable side effects. Much research now indicates that precision medicine can be used for HNSCC and may achieve improved results. From this perspective, we present an overview of the current status, potential strategies, and challenges of precision medicine in HNSCC. We focus on targeted therapy based on cell the surface signaling receptors epidermal growth factor receptor (EGFR), vascular endothelial growth factor (VEGF) and human epidermal growth factor receptor-2 (HER2), and on the PI3K/AKT/mTOR, JAK/STAT3 and RAS/RAF/MEK/ERK cellular signaling pathways. Gene therapy for the treatment of HNSCC is also discussed.

## INTRODUCTION

Precision medicine represents a new era in medicine­ - one that delivers the most appropriate treatment at the most appropriate time enabled, in large part, by the mapping of the human genome [1]. Its initial purpose is focused on cancer, by providing a new strategy for cancer treatment approaches.

Head and neck squamous cell carcinoma (HNSCC) is the sixth most common cancer all over the world. It remains a serious public health problem [2, 3]. More than 500,000 new head and neck cancer patients are diagnosed and approximately 300,000 deaths are caused by HNSCC every year. There have been 48,300 new cases and 9,570 deaths just in USA according to Rebecca L. Siegel's report for 2016 [2]. Over 90 percent of cancers that arise from the oral mucosa are diagnosed as squamous cell carcinomas [4]. HNSCC also remains a serious public health problem in Asian countries [5]. However, the current therapies including surgery, radiation therapy, Cisplatin-based chemoradiotherapy and pharmacotherapy are often aggressive [6, 7] and carry considerable side effects [8]. In most cases these treatments lead to poor prognostic outcomes [6]. Only 40%-50% of patients with HNSCC survive for 5 years [9]. When accurate diagnosis and improved prognosis are required for oral cancer [10], precision medicine gives the HNSCC patients new hope.

In this paper, we will summarize the main steps in the emergence of precision medicine, and then focus on how precision medicine is used in HNSCC, especially for targeted therapy and gene therapy.

## EMERGENCE OF PRECISION MEDICINE

**Figure 1:**
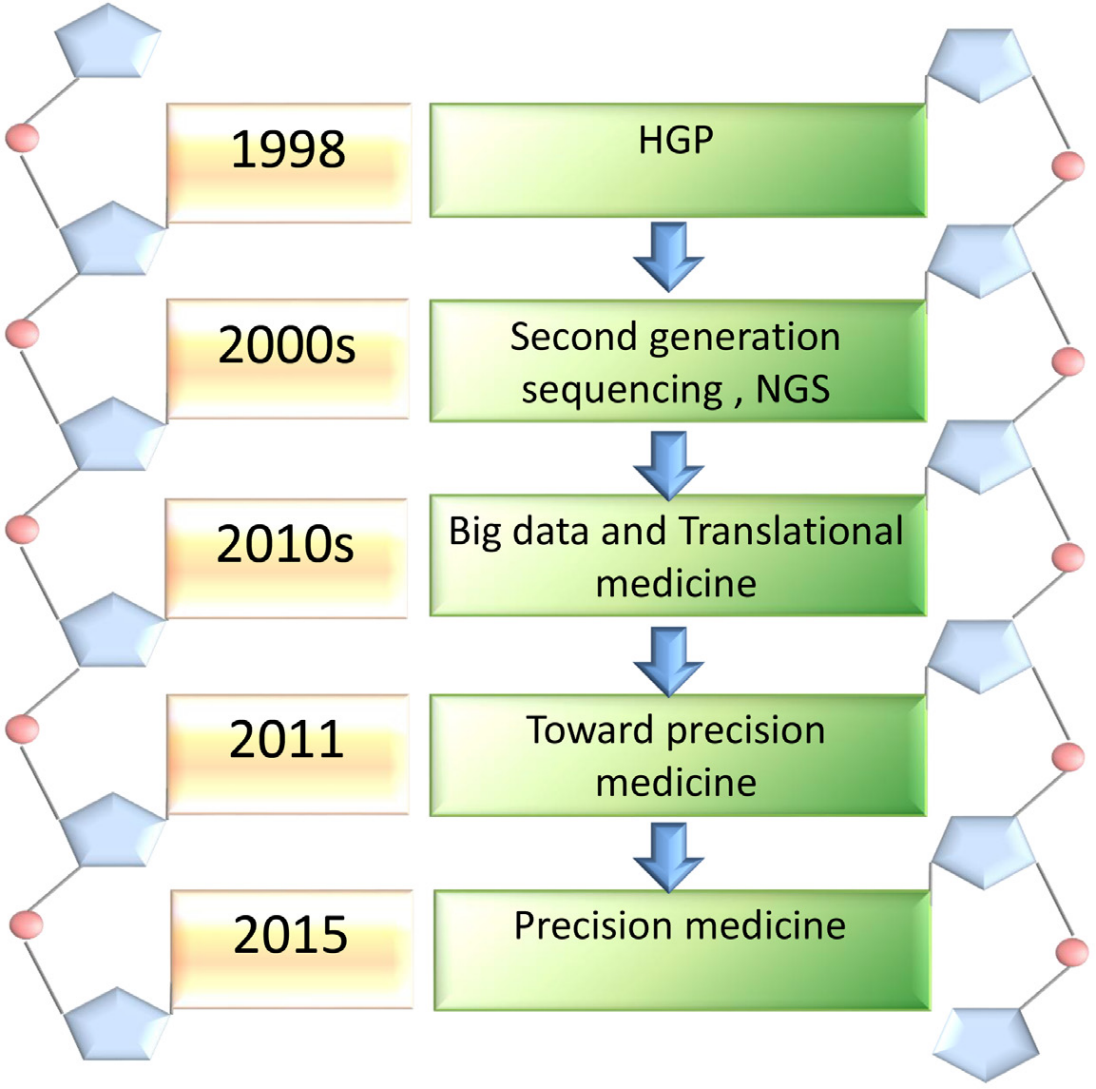
The major steps of the emergence of precision medicine Precision medicine experienced five important steps from 1985 to 2015. HGP leads us to enter the genomic era in 1985. With the explosive development of biomedical technology, the second and next generation sequencing technology was invented in the 2000s, making gene sequencing much faster, cheaper and preciser. Big data and translational medicne promoted the development of precision medicine. A framework of the “toward presision medicine” established a conception of new therapeutic strategy in 2011. Precison medicion was presented officially by President Obama in 2015.

Precision medicine is an emerging therapeutic strategy that aims at disease prevention and treatment through comprehensive analysis patient's individual variations in genes, environment, and lifestyle [11]. It is beyond the scope of P4 medicine (predictive, personalized, preventive and participatory) [12]. The term “precision” has largely replaced “personalized”, because it considers the patient and health care system interaction in a more accurate manner. Precision medicine is a personalized intervention therapeutic method based on the big OMICS data including epigenetics, genomics, proteomics and metabolomics, and is involved in the classification, staging and grading of the disease depending on histopathology or immunology. Precision medicine may be used to predict the development of the diseases and patients’ response to therapies, facilitating clinical decision making to achieve the best curative effect [13]. It contains two key factors: (1) building a biological database; (2) utilizing the data to guide the individual disease diagnose and treatment. Five important steps are involved in the process of proposition precision medicine (Figure 1).

It has been over forty years since man's first landing on the moon-Project Apollo. Human landed on the “moon” again-Human Genome Project (HGP), which was initiated in 1985 [14]. Five years later, HGP was launched with the approval of the congress of the United States, planning to spend at least $3 billion for the analysis of the human genome in 15 years. It was an important day when scientists who were from the United States, United Kingdom, France, Germany, Japan and China announced that they finished drawing the human genome sketches in 2000 [15]. The completion of HGP is an important step forward towards understanding the mysteries of the human beings.

With the completion of HGP and the rise of second-generation sequencing technology and next-generation sequencing technology (NGS) [16], bioinformatics analysis of large sets of data has been dramatically amplified in the 2000s. How should the big data be appropriately used for clinical applications? Translational medicine has partially solved this problem, but it is not sufficient [17, 18]. To address this question, the National Academy of Sciences made a systematic discussion of a new concept “Precision Medicine” in “Toward Precision Medicine: Building a Knowledge Network for Biomedical Research and a New Taxonomy of Disease” [19].

President Obama launched a new Precision Medicine Initiative, which aims to cure diseases like cancer based on using of personalized information to keep ourselves and our families away from diseases on January 30^th^, 2015. In his speech, he had infinite faith in that public health would significant progress with the development of scientific research. He established a research institution aiming to make the best use of genetically informed approaches for cancer treatment. Over 1 million volunteers are involved in this project. It is believed that it is time to realize his far-sighted initiative, and the National Institutes of Health (NIH) as the main leadership and other participators will join hands with each other to make precision medicine come true [20].

## APPLICATION OF PRECISION MEDICINE TO CANCER THERAPY

Cancer is one of the major diseases leading to death. Recent studies have demonstrated that cancer can be caused by a series of molecular damage and that each cancer has individual and characteristic genomic codes with both common and tumor-specific features [20]. NIH announced their near-team goals­ - intensifying efforts to apply precision medicine to cancer, including innovating clinical trials of targeted drugs, using combination therapies, and overcoming drug resistance. President Obama unveiled the Precision Medicine initiative during the State of the Union address. Focusing on cancer, it aims to encourage more volunteers to join this research and build biomedical data by collecting and dealing with the genotypic, phenotypic, environment and lifestyle information [21]. Cancer Medicine Program (CMP) aims to utilize precise genome sequencing and information analysis for accurate diagnosis of cancer, molecular typing, treatment response prediction and interpretation for resistance to cancer drugs. In fact, the discovery of the most effective targeted cancer therapies comes from cancer genetics and genomics studies. Accordingly, cancer genome sequencing may provide cancer patients with effective personalized precision therapies [23]. Numerous previous studies have demonstrated the power of precision medicine in cancer treatment [24, 25].

## PRECISION MEDICINE FOR HEAD AND NECK SQUAMOUS CELL CARCINOMA

### Precision prediction and diagnosis of HNSCC

#### Oral potential malignant disease (OPMD)

Oral potential malignant disease (OPMD), such as oral leukoplakia, erythroplakia, is likely to cause oral squamous cell carcinoma (OSCC) since more than 62 percent of OSCC results from OPMD [27]. Therefore, the identification of the underlying relationship between OPMD and OSCC should enable early diagnosis of OSCC. A large number of studies have been performed to explore the potential relevance between OPMD and OSCC [28]. Sequencing results of the lesions that have high risk of malignancy may be used to predict and improve disease progression and outcome.

In the study by Jay O. Boyle [29], the incidence of p53 mutations was detected in 19 percent of non-invasive lesions and 43 percent of invasive carcinomas, indicating that p53 mutations increased in preceding invasion in primary head and neck cancer. In the study by Guozhong Qin et al. [30], the p53 mutations were found in both erythroplakia lesions and oral squamous cell carcinoma. Sciubba James J indicated that the p53 mutations increase the incidence of erythroplakia in developing malignancy and proved that p53 mutation is an early event during the development of OSCC [31]. Davide B. Gissi found that the lesions express p53 and Ki67 at the same time, indicating that the non-dysplastic oral leukoplakia may be at risk of developing oral cancer through a retrospective longitudinal study [32].

Nilva K. Cervigne [24] found that the DNA copy numbers underwent unpredictable change in the process of DNA replication. Some of the copy numbers increased like 1p (over 80%), 11q13.4 (68%) and 9q34.13 (64%), especially the quite obvious increasing on the segment of 1p35 and 1p36. He also observed that other regions of DNA copy number were missing in more than 20 percent of samples, especially on 5q31.2 (about 35%), 16p13.2 (about 30%) and so on. In sum, Nilva K. Cervigne concluded that there is a correlation between the DNA copy number alterations (CNAs) and the different grades of dysplasia in 70 percent of patients. These statistical data indicated that CNAs may be connected with OSCC progression. Recent studies reached similar conclusion that the DNA copy number change (gain and lose) can be used to predict the risk of OSCC progress in patients with OPMD [33-35].

By examining microRNA expression in twelve patients with progressive leukoplakias and four with non-progressive leukoplakias, Cervigne NK found that 109 microRNAs were specifically overexpressed in patients with progressive leukoplakia and invasive OSCC. The malignant transformation might be closely related to the high-expression of microRNA-21, microRNA-181b and microRNA-345. His findings suggested that the change of microRNA expression can predict the risk of malignant transformation of leukoplakia [36]. Guanghui Zhu also provided direct evidence that microRNA played an important role in the development of leukoplakia to oral cancer [37]. It was reported that about 37.5 percent of microRNAs were related to oral cancer in previous literature [37].

#### Head and neck squamous cell carcinoma (HNSCC)

Oncogenes and tumor suppressor genes play critical roles in cancer initiation and progression. The dysregulated expression and mutation of these genes are detected in all kinds of cancers and the related studies have been fruitful in the past decades. Oncogenes and tumor suppressor genes are now frequently used as molecular targets for cancer treatment [38]. Genetic alterations are considered as an important step in the development and progression of HNSCC, leading to the dysregulation of essential cellular signaling pathways involved in cell proliferation and differentiation [39]. Progressive allelic loss is one of the significant steps in the development of HNSCC. There have been many oncogenes and tumor association suppressor genes identified in the previous studies, including p53 [40], p16 (CDKN2A) [41, 42], NOTCH, EGFR [43], RAS, CCND1 [44], BRAF, and PIK3CA [45, 46]. The roles of these genes and their therapeutic potential in HNSCC have just begun to be revealed [26, 47-49]. The ratios of mutation or dysregulated expressing of key genes are listed in Table 1.

**Table 1:** Application of key genes and their ratios of mutation or expressing

Gene	Ratios of mutation/ expressing	Application	Refs
P53	50% mutation	Precision prediction and diagnosis; Gene addition therapy; Precision surgery	[35, 51, 52, 123]
EGFR	95% overexpressing	Precision prediction and diagnosis; Targeted therapy; Gene disruption therapy; Targeted radionuclide therapy	[72, 75-84, 125]
VEGFR	33% overexpressing	Targeted therapy; Gene disruption therapy; Targeted radionuclide therapy	[79, 81, 88, 126]
HER2	1%-2% overexpressing	Targeted therapy	[93, 95, 98, 99]
NOTCH1	10%-18.6% mutation	Precision prediction and diagnosis	[55, 56]
PI3K/Akt/mTOR	30.5% mutation	Targeted therapy	[101-103]
RAS/RAF/MEK/ERK	6% mutation	Precision prediction and diagnosis; Targeted therapy	[38, 71, 105]
JAK/STAT3	Rarely mutated	Targeted therapy	[107, 108]
CDKN2A	78.7% overexpressing	Precision prediction and diagnosis	[43]

The ratios of mutations or expressing of several important genes are shown by this table. The table also demonstrates how gene targets are used in precision medicine.

As a tumor suppressor gene, p53 gene contributes to the arrest of tumor cell proliferation and tumor growth [50, 51]. P53 gene mutation can be detected in about 50 percent of HNSCC patient samples [50, 51]. The incidence of p53 mutation significantly increased when the pre-malignant diseases progressed to invasive cancer [52, 53].

NOTCH1 is another important tumor suppressor gene. Approximately 10 to 18.6 percent of HNSCC patients carry mutations in the NOTCH1 gene, making NOTCH1 the second most frequently mutated gene next to p53 [54, 55]. Nicolas Stransky's findings indicted that the dysregulation of NOTCH signaling enhanced the genesis and progression of HNSCC [3].

It was found that HPV infection is an important risk factor associates with a large group of HNSCC patients [56]. HPV-positive and HPV-negative HNSCCs have distinct clinical features and dissimilar biology [9, 57]. Approximately 22-26 percent of HNSCCs were found to be HPV positive. The prevalence was even up to 35-41 percent in OSCCs [57, 58]. The treatment outcome of the HPV-positive patients was deficient [59]. It may be associated with HPV oncoproteins E6 and E7 [58].

Telomerase is also an important marker for early diagnosis and prognosis of cancer, including HNSCC [60, 61]. Promoter hypermethylation has been found to be related to the assessment of HNSCC patients’ prognosis. Examination of the hypermethylation status can be used for early diagnosis, invasive detection and tumor surveillance in clinic [62, 63].

### Precision medicine for the treatment of HNSCC

**Figure 2:**
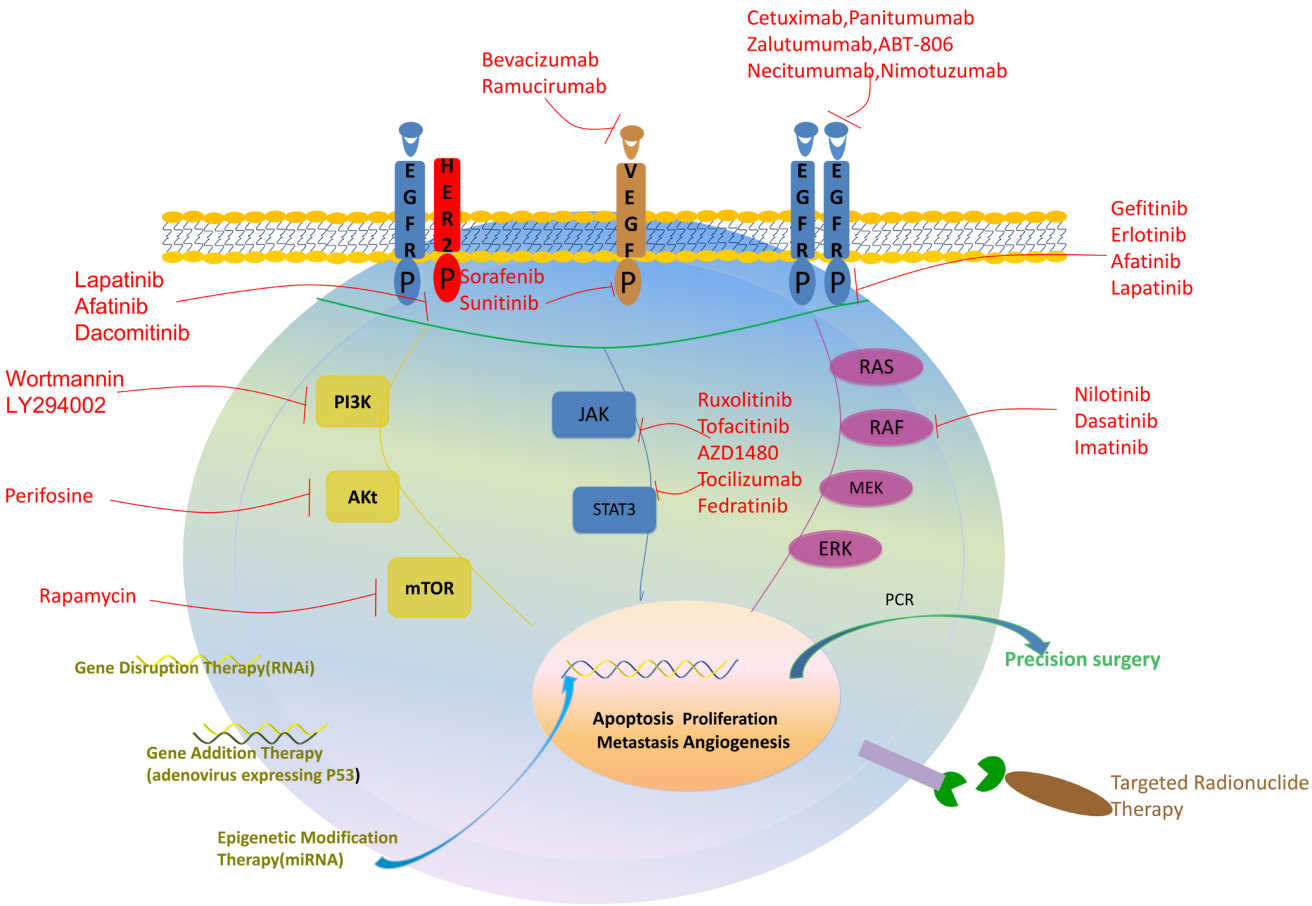
Precision medicine used for the treatment of HNSCC This figure shows 5 major methods of precision medicine used in HNSCC patient: (1) inhibitors targeting cell surface signaling receptors, including EGFR, VEGFR, HER2 (2) inhibitors targeting cellular signaling pathways, including PI3K/Akt/mTOR, RAS/RAF/MEK/ERK, JAK/STAT3 (3) precision genetic manipulation therapies, including gene disruption therapy, gene addition therapy and epigenetic modification therapy (4) precision medicine combining with surgery (5) precision targeted radionuclide therapy.

Traditional therapeutic modalities such as surgery, chemotherapy, and radiation therapy have their own limitations [17, 64, 65]. Advanced understanding of the molecular mechanism of cancer has highlighted targeted therapies as more promising cancer therapeutics for patients with HNSCC (Figure 2).

#### Targeting cell surface signaling receptors

EGFR

Epidermal growth factor receptor (EGFR), a cell surface receptor, is one of the most important targets for HNSCC. EGFR is a member of the ErbB growth factor receptor tyrosine kinase family, which has four members: EGFR-ErbB1, Her2-neu-ErbB2, HER3-ErbB3 and HER4 -ErbB4 [7, 66-68]. Hyperactivated EGFR signaling has been frequently found in HNSCC cells. EGFR is involved in the regulation of cell proliferation, metastasis, migration, invasion, angiogenesis, and inhibition of apoptosis [69-71]. Although rarely mutated, EGFR is highly upregulated in many cancers with epithelial origins, including HNSCC in which EGFR upregulation is found in over 95% of the tumors [72]. There are many approaches used to inhibit or downregulate the EGFR, such as tyrosine kinase inhibitors (TKls), monoclonal antibodies (mAbs), ligand-linked toxins, and antisense oligonucleotides [67, 73-75]. Two of them are most widely used in clinical models: (1) monoclonal antibodies (mAbs) targeting extracellular surface receptor, including cetuximab, panitumumab, zalutumumab, ABT-806, necitumumab and nimotuzumab; (2) tyrosine kinase inhibitors (TKIs) functioning as a competitor of ATP, including gefitinib, erlotinib, afatinib and lapatinib [76-84]. Cetuximab is the only FDA approved molecular targeted agent in HNSCC, and it has a significant effect combined with radiotherapy, chemotherapy or radio-chemotherapy [85, 86]. Although a case was reported that using cetuximab as a single agent got ideal outcome, the current data did not provide solid evidence for its use as a monotherapy drug [86, 87]. Zalutumumab extended progression-free survival of patients with recurrent HNSCC and median overall survival was increased from 5.2 months to 6.7 months [78]. The patients with HNSCC were assessable for response to TKIs, with an observed response and a disease control, but further study is needed for the better understanding of their clinical application [76, 84, 85].

HER2

Human epidermal growth factor receptor-2 (HER2) is a member of the epidermal growth factor receptor family and activates a variety of signaling pathways, leading to cell proliferation and tumorigenesis [88, 89]. HER2 has been found to be overexpressed in a number of human cancers, including HNSCC, contributing to tumor development, cell cycle progression, cellular motility and growth [90]. A number of strategies are used to inhibit HER2, including mAbs and small-molecule TKIs. Trastuzumab and pertuzumab are the most successful mAbs [91]. Lapatinib, afatinib and dacomitinib are also effective therapeutic agents targeting HER2 [88, 92, 93].

VEGFR

Vascular endothelial growth factor (VEGF) is the most effective pro-angiogenic growth factor, which regulates the growth of blood vessels. VEGF has been identified as an independent prognostic factor for HNSCC [94, 95]. Angiogenesis is critical for tumor growth and metastasis, and becomes an important target of anticancer treatment. Anti-angiogenesis agents have shown promising therapeutic effect on several solid tumors including HNSCC [96], such as bevacizumab, bamucirumab, sorafenib and sunitinib. A combination of anti-angiogenesis agents with other agents achieved enhanced efficacy for HNSCC treatment [97-99].In Maria Vassilakopoulou's study, it was reported that 3-year progression-free survival rate was improved from 60% to 86% in patients using bevacizumab combining with other antitumor therapies [96].

#### Cellular signaling pathways

PI3K/Akt/mTOR

Recent progress in understanding the oncogenesis of HNSCC has revealed multiple dysregulated signaling pathways [100]. One frequently altered axis is the PI3K/Akt/mTOR pathway observed in 30.5 percent of HNSCC patients [101-103]. It plays a crucial role in HNSCC progression. PI3K/Akt/mTOR pathway is involved in essential cellular functions including cell proliferation, apoptosis and differentiation. Wortmannin (a natural product) and LY294002 (a synthetic drug) are two representative first-generation PI3K inhibitors whose application has so far been primarily restricted to preclinical studies [100]. A well-established lipid-based Akt inhibitor, perifosine showed potent activity in the inhibition of cell proliferation in preclinical studies [104]. The first mTOR inhibitor rapamycin, a natural product, was originally used for its antifungal properties before its immunosuppressive and antineoplastic effects were discovered [100]. Enhanced efficacy was achieved when these agents were used in combination with other drugs (cetuximab, cisplatin, etc.) in patients with HNSCC [102].

RAS/RAF/MEK/ERK

RAS/RAF/MEK/ERK pathway is another major signaling pathway for HNSCC cell survival [71]. Most often used RAF inhibitors are nilotinib, dasatinib, and imatinib [105]. So far, however, the clinical potential of RAF inhibitors in HNSCC remains to be further explored [38].

JAK/STAT3

JAK/STAT pathway is a newly discovered signal transduction pathway which contributes to the proliferation and survival of tumor cells including HNSCC [106-108]. Several JAK/STAT kinase inhibitors have been identified and are currently being tested in clinical trials, including ruxolitinib, tofacitinib, AZD1480, tocilizumab, fedratinib and pyrimethamine, due to their contribution to treatment resistance and immune escape. [107]. AZD1480 was demonstrated to inhibit proliferation of cell lines andpatient-derived xenograft in HNSCC preclinical models [106].

#### Precision gene therapy

Gene disruption therapy

Gene disruption therapy is an effective approach in decreasing tumor cell proliferation, slowing tumor invasion and reducing drug resistance. RNA interfere technique (RNAi) was successfully used to target and disable a group of crucial genes for HNSCC treatment [109, 110], including EGFR [111], EpCAM [112], MET receptor [113], Cyclin D1, NF-κB, p65, VEGFR, telomerase reverse transcriptase and p63 [111].

Gene addition therapy

Gene addition therapy is a therapy of adding specific genes to tumor cells with lower or absent expression of such genes. Gene addition therapy has demonstrated its potential anti-tumor effects for HNSCC treatment. Gene addition therapy targeting p53 gene has been very well established [110, 114]. The treatment approaches of delivering p53 have been tested in HNSCC by direct injection of an adenoviral vector expressing wild-type p53 gene [115]. In Peng's report [116], radiotherapy in combination with p53 addition therapy showed a dramatically improved efficacy (64% showing complete response) than using radiotherapy alone in more than 2000 patients with advanced HNSCC.

Epigenetic modification therapy

The differential expression of miRNAs between cancer and normal tissue has been well documented. Specific miRNAs expression was detected in HNSCC cells [118]. MiRNA involves in a variety of regulatory pathways, including cell proliferation, apoptosis and metabolism. Approaches specifically targeting on miRNAs demonstrated significant anti-cancer potential [118].

#### Precision medicine in combination with surgery

Most surgeons rely on histological examination like frozen biopsy to determine the surgical margins. However, the local recurrence of HNSCC is often reported in clinical practice [119, 120]. Therefore, a new method to determine the surgical margins is urgently needed. From the perspective of molecular biology, detecting mutation genes or abnormal protein to find rare cancer cells provides a new strategy [120-122]. Polymerase chain reaction (PCR) or immunohistochemistry can be used to detect rare cancer cells. Examination of p53 mutations has been used to detect such cells [35, 122]. Precision surgery with molecular detection can decrease the incidence of local recurrence.

#### Precision targeted radionuclide therapy

Targeted radionuclide therapy (TRT) is a new therapy of cancer with using the targeted bridging drugs to improve the sensitivity of ray to the tumor cells, which causes less collateral damage to normal tissues. Targeted bridging drugs that play a crucial part in the TRT have two connection ends. One of the ends carries radionuclides and the other shows high afﬁnity to the surface antigens of tumor cell [123, 124]. EGFR mutation is most often observed in HNSCC and considered as a suitable target for TRT [125]. VEGF is also a potential TRT target [80, 82, 126].

## FUTURE AND CHALLENGES

Big data does not directly translate genetic information into clinically useful interventions to benefit HNSCC patients [54, 127]. One of the limitations is insufficient large-scale genome sequencing for HNSCC. It's necessary to build database of HNSCC with larger numbers of patients. In addition, the sharing of data is limited among different countries. Electronic health records will be essential for communication among providers, patients, and researchers regarding crucial medical information [11]. A better designed platform for appropriately sharing clinical information is needed.

There are very few desired solutions for drug resistance [128, 129]. The patients with drug-resistance can be better classified to identify their commonalities by using sequencing technology. With the help of precision medicine, delays in the patient's diagnosis may be avoided, and more suitable drugs may be chosen.

Combination of anti-tumor therapies may lead to improved treatment outcome, but the underlying mechanism is poorly understood [130-132]. In the ongoing studies, the unknown tumor molecule and genetic pathogenesis will be identified to provide new precision therapeutic targets [8, 126].

## CONCLUSIONS

Precision medicine raises everyone's hopes. Precise treatment is based on gene sequencing, proteomics, genomics and molecular biology. Ideally, each patient should receive a unique optimized treatment regimen based on differentially expressed genes and proteins to achieve the best clinical outcome. Precision medicine gives us a new strategy with which to pursue a cure for HNSCC. Further understanding of the underlying molecular mechanisms will lead to the development of HNSCC therapies with significantly enhanced efficacy.
